# At same leucine intake, a whey/plant protein blend is not as effective as whey to initiate a transient post prandial muscle anabolic response during a catabolic state in mini pigs

**DOI:** 10.1371/journal.pone.0186204

**Published:** 2017-10-18

**Authors:** Aurélia Revel, Marianne Jarzaguet, Marie-Agnès Peyron, Isabelle Papet, Noureddine Hafnaoui, Carole Migné, Laurent Mosoni, Sergio Polakof, Isabelle Savary-Auzeloux, Didier Rémond, Dominique Dardevet

**Affiliations:** Université Clermont Auvergne, INRA, UNH, Unité de Nutrition Humaine, PFEM, MetaboHUB-Clermont, CRNH Auvergne, Clermont-Ferrand, France; Deakin University, AUSTRALIA

## Abstract

**Background:**

Muscle atrophy has been explained by an anabolic resistance following food intake and an increase of dietary protein intake is recommended. To be optimal, a dietary protein has to be effective not only to initiate but also to prolong a muscle anabolic response in a catabolic state. To our knowledge, whether or not a dairy or a dairy/plant protein blend fulfills these criterions is unknown in a muscle wasting situation.

**Objective:**

Our aim was, in a control and a catabolic state, to measure continuously muscle anabolism in term of intensity and duration in response to a meal containing casein (CAS), whey (WHEY) or a whey/ plant protein blend (BLEND) and to evaluate the best protein source to elicit the best post prandial anabolism according to the physio-pathological state.

**Methods:**

Adult male Yucatan mini pigs were infused with U-^13^C-Phenylalanine and fed either CAS, WHEY or BLEND. A catabolic state was induced by a glucocorticoid treatment for 8 days (DEX). Muscle protein synthesis, proteolysis and balance were measured with the hind limb arterio-venous differences technique. Repeated time variance analysis were used to assess significant differences.

**Results:**

In a catabolic situation, whey proteins were able to initiate muscle anabolism which remained transient in contrast to the stimulated muscle protein accretion with WHEY, CAS or BLEND in healthy conditions. Despite the same leucine intake compared to WHEY, BLEND did not restore a positive protein balance in DEX animals.

**Conclusions:**

Even with WHEY, the duration of the anabolic response was not optimal and has to be improved in a catabolic state. The use of BLEND remained of lower efficiency even at same leucine intake than whey.

## Introduction

Skeletal muscle size and function is directly related to the amount of muscle protein content and quality. Muscle protein homeostasis is ensured by a continuous turn-over including degradation of non-functional muscle proteins which are replaced by the synthesis of new ones. This equilibrium between muscle protein synthesis and breakdown varies throughout the day. During the post-absorptive period, in absence of food intake, muscle proteolysis exceeds synthesis in order to provide essential amino acids to other organs whereas, an anabolic response is initiated with a muscle protein synthesis higher than proteolysis after food intake [1]. In healthy adults, on a day-to-day basis, the rate of muscle protein breakdown is then tightly balanced by equal rates of muscle protein synthesis in order to ensure the maintenance of muscle size and optimal muscle functions over time [2]. It is now well established that the post prandial anabolic response of skeletal muscle is explained by the stimulation of protein synthesis initiated by the increased essential amino acids bioavailability whereas the decrease in proteolysis is attributed to the stimulation of insulin secretion in response to feeding [3, 4]

Even if decreased physical activity has been identified as a cause, muscle atrophy has also been explained by the presence of an anabolic resistance following food intake in several physio-pathological catabolic states as diverse as aging, cancer, disuse, advanced obesity [5–12], or in cases of reductions in limb blood flow and skeletal muscle microvascular perfusion [13]. The cause of this anabolic resistance is multi factorial but the alterations of muscle protein metabolism response to food intake have been partially explained by the presence of a low grade inflammation, an oxidative stress, a lipotoxicity, an elevated glucocorticoid level or an increase of splanchnic extraction of dietary amino acids [14–22] In all cases, an increase of dietary protein intake above the current recommended dietary allowances (RDA at 0.8g/kg/d) [23] has been recommended to overcome the anabolic resistance observed. Nevertheless, the biological value of proteins may vary among the dietary sources, and then this aspect must also be considered in terms of amino acid composition and digestion/absorption kinetics which influence plasma bioavailability of amino acids and then their ability to initiate an optimal muscle anabolic response. Among dietary proteins, fast digested and leucine rich proteins (i.e whey) have been shown to be the most effective to stimulate muscle protein synthesis, presenting positive effects during aging or disuse when compared to proteins with lower leucine content and whose digestion rate is slower [24–26]. However, more inconsistent results have been found in other anabolic resistance states such as cancer- or glucocorticoids treatment-associated muscle wasting [27–29]. These discrepancies could be explained by the nature and intensity of the catabolic state but also by the fact that muscle protein synthesis was often assessed at a specific time after the protein intake. Muscle protein anabolism is indeed a non steady state response to food intake for which the intensity and duration is dependent of the type and amount of the protein ingested but also influenced by the physiopathological state ([30]). Then it is necessary to assessed muscle protein anabolism continuously over the full anabolic response in order to conclude on the efficiency or inefficiency of a given protein source in an anabolic resistance situation.

The different properties of the various dietary proteins can also be used if they are given in the form of mixtures. This has mainly been studied in sport nutrition to optimize muscle anabolism following exercise. Indeed, Butteiger at al have shown in rats that a dairy/plant protein blend extended the duration and intensity of the post prandial skeletal muscle protein synthesis when compared to whey proteins [31]. The same team also showed that a dairy/plant protein blend was able to stimulate muscle protein accretion following resistance training in humans which was also slightly prolonged compared with whey [32–34]. To our knowledge, whether or not a dairy/plant protein blend could be as effective or more effective to initiate and prolong the muscle anabolic response in a catabolic state is still unknown.

In a hindlimb multi-catheterized minipig model, the aim of the present study was to measure continuously (over the entire fed state), muscle protein synthesis and proteolysis before and after the ingestion of a complex meal containing casein or whey or a dairy/plant protein blend containing the same leucine content. The potential differential effect of these various proteins supplies were also tested in a catabolic state induced by a glucocorticoids treatment, a very well characterized model known to induce an anabolic resistance.

## Materials and methods

### Animals housing, surgery and ethics statement

The present study was approved by the Animal Care and Use Committee of Auvergne (CEMEA Auvergne; Permit Number: CE 68–12) and the Ministère de l’Enseignement Supérieur et de la Recherche (n° 02125.02). For the study, 36 adult maleYucatan mini pigs (averaging 20kg) have been housed individually in subject pens (1 X 1.5 m) in a ventilated room with controlled temperature (21°C). They were fed twice daily with 220 g/d of a concentrate feed containing 16% protein, 1% fat, 4% cellulose and 5% ash (Porcyprima; Sanders Centre Auvergne, Aigueperse, France) and had free access to water.

Three weeks before the experimentation, minipigs went into surgery. Before surgery, the animals were fasted 24h. Half an hour after premedication by intramuscular injection of 7 mg/kg ketamine (Imalgène 1000®; Rhône Mérieux, Lyon, France), 1.5 g/kg azaperone (Sresnil®, Janssen Pharmaceutica, Beerse, Belgium) and atropine (Atropine 0,1%, Laboratoire Aguettant, Lyon, France), deep anesthesia was induced, via snout mask, with 10% isoflurane (Forene®, Abbot, Queenborough, UK) carried by oxygen, for 10 min. After oral endotracheal intubation, isoflurane concentration was reduced to 2% in order to maintain general anesthesia during the surgery. After median ventral laparotomy, minipigs were fitted with catheters (polyvinyl chloride; 1.1-mm ID, 1.9-mm OD) in the hepatic vein, the inferior caval vein, and the aorta. The hepatic vein catheter was placed via a small incision on the left lobe of the liver, with the tip positioned 1–2cm before the caval vein (checked by palpation). For the caval vein, the catheter was inserted just downstream the junction of the ileac veins, using a needle, and pushed over 3–4 cm in the vessel, with the tip positioned 3–4 cm before the affluence of the renal veins. A transit time ultrasonic blood flow probe (6-mm probe, R-series; Transonic Systems, Inc., Ithaca, NY) was implanted around the distal aorta1-2 cm before it splits in the iliac arteries. Catheters and probe cables were exteriorized through the skin of the right flank of the animal. The abdominal incision was closed in three layers. After surgery, minipigs were fitted with a canvas harness (Lomir Biomedical, Malone, NY) to protect catheters and probe connectors. The first 4 days after surgery, minipigs were treated with an antibiotic (Clamoxyl, GlaxoSmithKline) as a prophylactic measure, and Flunixin (Finadyne®, Shering-Plough, Uxbridge, UK) was administered intravenously for postoperative analgesia. Feed allotments were progressively increased over 7 days until the pre-surgery daily ration. A minimum of 2 weeks was allowed for recovery from surgery before initiating the experiment. Surgical procedures, as well as post-surgical care, have been previously described in detail by Rémond et al. [35]

### Tracer infusion studies.

**Fig 1:**
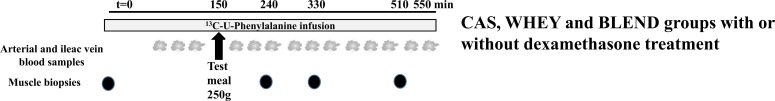
Schematic of the protocol. Animals (n = 6) were studied either in normal condition or after a glucocorticoid treatment (dexamethasone). After fasting, they were infused with ^13^C phenylalanine and received a mixed meal (t = 150 min) with casein (CAS) or whey (WHEY) or whey/plant proteins (BLEND). From 90 min to 550 min, blood samples were withdrawn every 30 minutes.

The day of the experiment, after an overnight fasting period, animals were separated into 3 groups (n = 6). After basal blood samples were withdrawn from the artery and ileac vein, a priming dose of labeled [ring U-^13^C] L-Phenylalanine (4.2 μmol.kg^-1^) was injected and then [ring U-^13^C] L-Phenylalanine was infused (4.2 μmol.kg^-1^.h^-1^) for 550 min through the hepatic vein. During the first 150min, animals remained deprived of food and samples of arterial and ileac venous blood were simultaneously withdrawn at 90, 120 and 150 min and represented the post-absorptive period (PA)(**Fig 1**). At t = 150min, animals consumed a test meal (250g) containing as protein sources either casein (CAS), whey (WHEY) or whey/plant proteins (BLEND)) (Table 1). The meal was entirely consumed within 10–15 min, arterial and ileac venous blood was sampled every 30 min during the remaining 400 min (post prandial period, PP) **(Fig 1**). The casein and whey test meals contained the same amount of dietary proteins (13%) but differed in term of leucine content (+38% whey vs casein) and digestion speed characteristics ([21, 30]. The whey/plant protein meal was composed of 70% pea and wheat protein sources (Roquette, France) in a ratio of 2.5 to 1 in order to re-equilibrate the amino acid content in lysine and sulphur amino acids (Table 1). To adjust the leucine intake to the whey meal, the whey/plant protein test meal also contain 30% of whey and its protein content has been increased at 16.5%. Then, the plant based protein test meal allowed a substitution of animal proteins by 70% but remained identical to the whey meal in terms of leucine intake (**Table** 1). We used whey proteins instead of free leucine to increase leucine content of the protein blend because we showed in a previous study that free leucine supplementation over a casein protein source remained ineffective compare to whey in a model anabolic resistance recovery after disuse [12]. Blood flow was recorded continuously throughout the whole experiment with the ultrasonic blood flow probe.

**Table 1:** Composition of the experimental mixed meals.

Gram	CAS 13%	WHEY 13%	BLEND 16.5%
**Casein Ca**^**2+**^	**157.2**	**0**	**0**
**Whey**	**0**	**145.6**	**58.9**
**Pea Proteins**	**0**	**0**	**100.6**
**Wheat Proteins**	**0**	**0**	**40.2**
**Cystine**	**1.5**	**0**	**0**
**Proline**	**0**	**4.7**	**0**
***Leucine content***	**11.7**	**16.2**	**16.1**
***Total EAA content***	**74.1**	**78.3**	**84.0**
**Lactose**	**4.7**	**0**	**4.9**
**Rapeseed oil**	**30**	**30**	**30**
**Sunflower oil**	**3**	**3**	**3**
**Peanut oil**	**27**	**27**	**27**
**Cellulose**	**35**	**35**	**35**
**Sucrose**	**100**	**100**	**100**
**Wheat starch**	**641.6**	**654.7**	**600.3**

Foot note: Protein sources: casein (calcium caseinate, isolate, 90% N2 (Lactalis, France), Whey (Native proteins, isolate, 90%N2 (Lactalis, France); Pea proteins (concentrate, 80%N2, Roquette, France); Wheat (concentrate, Roquette, France)

Following this experimental day, animals were allowed to recover for a week and returned to their usual feed. The glucocorticoid treatment (dexamethasone) was then started at a dose of 0.4mg/kg/day added to the feed every morning (0.15mg/kg) and evening (0.25mg/kg). Food intake was not modified by the treatment. After 8 days of treatment, the animals (n = 6 per test meal) underwent the same tracer infusion protocol described above except that dexamethasone was consumed with the test meal.

### Muscle biopsies and signalling

On a separate set of minipigs (n = 6 /test meal with or without dexamethasone treatment), a hindlimb muscle biopsy was performed at the fasted state and 90, 180 and 360 min after the test meal ingestion. These times corresponded to t = 150, 240,330 and 540 min in the tracer infusion protocol. Briefly, animals were intravenously injected with 5 mg/kg ketamine (Imalgène 1000®; Rhône Mérieux, Lyon, France) in order to induce anesthesia. Then, the sampling area (top of thigh) was disinfected with povidone iodine (Vétédine® solution 10%; Vetoquinol, Lure, France) and a locoregional anesthesia was performed by subcutaneous injection of 20 mg of lidocaine hydrochloride monohydrate (Lurocaine® 20mg/mL; Vetoquinol, Lure, France). An incision (1 cm) was made using a scalpel blade to cut the skin and the muscle fascia and 100 mg of muscle (femoral biceps) was sampled with a disposable core biopsy instrument (Bard®-Monopty®, 12G, 10 cm, Bard Biopsy System, Tempe, USA). Then, the incision was closed with a clip. The animal remained sedated for about 10min.

The muscle biopsy was immediately homogenized in 10 vol of buffer as previously described [36]. The homogenate was centrifuged at 10,000xg at 4°C for 10 min. Aliquots of supernatants were diluted in sample buffer, boiled for 5 min, and stored at -20°C until protein immunoblot analyses. Equal amounts of proteins were separated by SDS-Page (Mini-PROTEAN® TGX™ gels; Biorad; Marne la Coquette, France) and transferred to PVDF membranes using a Western blotting transfer system (Trans-Blot® Turbo™ Transfer System; Biorad; Marne la Coquette, France). Immunoblotting was performed using appropriate antibodies: Akt, p-Akt (T308), S6K1, p-S6K1(T389), S6, p-S6 (S240/244) (Cell Signaling, Beverly, MA, USA, lots 17, 16, 15, 15, 5, 9, respetively). On the same PVDF membrane, phosphorylated proteins were first assessed, then it was stripped and reprobed for the total protein. The blots were revealed using the Li-Cor Odyssey (Li-Cor Biosciences; ScienceTec, Courtaboeuf, France) procedure. Identified bands were quantified by densitometry using Odyssey software (Odyssey V3.0; ScienceTec, Courtaboeuf, France). Signals were corrected at same protein load assessed by the Stain Free Gels Technology during quantitation (BIORAD).

### Samples analysis

The plasma ^13^C enrichment of phenylalanine was measured by gas chromatography–mass spectrometry (GC-MS, model HP5975C/7890A, Agilent, Santa Clara, USA) with the use of tertiary-buthyldimethylsilyl derivatives and by monitoring the ions with m/Z 336 and 342 as previously described [14]. Briefly, 400 μL of plasma were homogenized in 8 volumes of ice-cold 10% trichloroacetic acid (TCA) and then centrifuged at 5000 x g for 15 min at 4°C. The resultant pellets (TCA insoluble materials) were washed 2 times in 4 volumes of 10% TCA. The combined supernatants, which contain free amino acids, were purified by cation exchange chromatography (AG 50 X 8, 100–200 mesh, H+ form, Biorad, Richmond, CA, USA) in minidisposal columns. Phenylalanine and other amino acids were eluted with 4M NH_4_OH. After evaporation of NH_4_OH under vacuum, free amino acids were resuspended in 0.01 M HCl for subsequent derivatization.

Plasma concentrations of amino acids were determined by ion exchange chromatography on deproteinized samples. An aliquot of plasma (700 μL) was homogenized in 7 volumes of trichloroacetic acid (0.6 mM) containing 2.5% of thiodiglycol. Norleucine (2.9 mM) was added as internal standard. Samples were incubated on ice for 20 min and centrifuged at 8000 g for 15 min at4◦C. This procedure was repeated once and pooled supernatants were passed through columns of cation exchange resin (AG 50W-X8, 100–200 mesh, Bio-Rad, Richmond, CA, USA). Purified amino acids eluted from the column by 4 mM NH4OH were dried and reconstituted in 1ml of 0.1mM lithium acetate buffer, pH 2.2. Amino acid concentrations were then determined using an automated amino acid analyzer (HPLC System, HITACHI).

Plasma glucose was assayed with an enzymatic method on an autoanalyser (Pentra 400, Horiba, and Montpellier, France) and insulin concentration was assayed by ELISA (Mercodia, Uppsala, Suède).

### Calculations

Muscle protein balance, protein synthesis and proteolysis have been calculated from arterio-veinous differences method according to the equations previously described by Bruins et al. [37] and Paddon-jones et al. [38]. Phenylalanine was selected to represent amino acid kinetics because it is neither produced nor metabolized in skeletal muscle [39]. In this tissue, the disposal and production of phenylalanine are a reflection of protein synthesis and protein breakdown.

The substrate net balance (NB) in nmol /kg/min was calculated as follow:

NB=Plasmaflow×[(AAA)−(AAV)]

where (AAA) and (AA_V_) are the plasma concentrations of the amino acid (μmol/L) in the arterial and ileac vein blood, respectively. Therefore, a positive NB represents net influx and negative NB represents a net efflux of substrate across the hindquarter.

The NB of the tracer in nmol /kg/min was calculated similarly according to the enrichment of phenylalanine (E%) in both arterial and ileac vein:

TracerNB=Plasmaflow×[(PheA×E%A)−(PheV×E%V)]

where (Phe_A_) and (Phe_V_) are the plasma concentrations of phenylalanine (μmol/L) in the arterial and ileac vein blood, respectively and E%_A_ and E%_V_ are the enrichments of Phe in the arterial and ileac vein plasma corrected with pretracer infusion values, respectively.

The disposal rate, i.e muscle protein synthesis, in nmol /kg/min was calculated as follow:

Proteinsynthesis=TracerNB÷E%V

where E% _V_ was thought to approach best the intracellular enrichment (precursor pool) of the organ [37, 40].

Because the NB of an amino acid across an organ is the net difference between production and disposal, the production, i.e proteolysis, is calculated as followed:

Proteolysis=Proteinsynthesis−SubstrateNB

Integrated muscle protein balance has been calculated for the following periods of time: post absorptive period (PA) (0–150min), early post prandial period (PP) (150–300min) and late post prandial period (PP) (300–550min). The time 300 min has been chosen because most of the studies assessing muscle protein synthesis with a single measurement are preformed 2–3h after food intake and because 300 min had been chosen in the present study to perform the energetic bolus.

$$Integrated\;muscle\;protein\;balance=\int_{t2}^{t1}NB÷(t2-t1)$$

### Statistics and analysis of results

Data are presented as mean ± SE. Given the complexity of the experimental design, statistical analysis was performed step by step, in order to compare the measured parameters over the post prandial period: comparisons of the 3 diets in treated or untreated animals, comparisons of DEX treated and untreated animals fed the same diet, comparisons of protein synthesis and protein degradation for a same diet and treatment. For this, repeated time variance analysis was used: variables introduced in the models were time (0 to 550 min), and either diet (CAS, WHEY, BLEND), either treatment (Control, DEX), either measurement (protein synthesis, protein degradation) and the corresponding interaction with time. At each time point, means were compared using either Tukey test (for 3 means to compare) or Fischer test (for 2 means to compare) as post-hoc tests. For glucose, insulin, protein synthesis, protein degradation and muscle protein balance, area under the curve were calculated and presented as histograms, but the same statistical analysis were used (repeated time variance analysis). All data are assessed for normality before the ANOVA analysis and the limit for statistical difference was set at P<0.05.

## Results

### Plasma glucose and insulin

**Fig 2:**
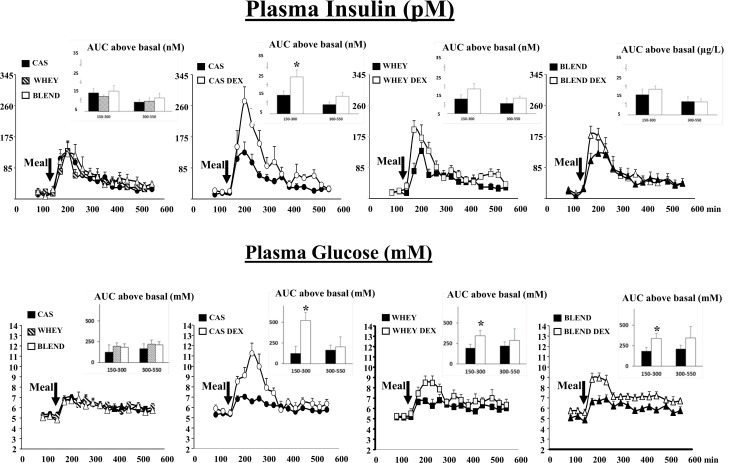
Plasma insulin and glucose in control and glucocorticoid-treated (DEX) mini pigs receiving either a casein (CAS), whey (WHEY) or whey/plant proteins (BLEND) mixed meal. The area under the curve (AUC) are presented in inserts. Post prandial AUC were calculated by subtracting the post absorptive values (t = 90–150min) to each post prandial values. Data are presented as means ±SEM. (n = 6). Repeated time variance analysis were used and at each time point, means were compared using Fischer test (for 2 means to compare). The limit for statistical difference was set at P<0.05. _*_ P<0.05 vs the control situation at the same time.

In the control groups, the effect of the ingestion of the three proteins sources on the plasma insulin and glucose levels was described and compared (first panel).After food intake, an increase of both insulin and glucose was observed in the control animals (**Fig 2**). Insulin was secreted rapidly with a maximum stimulation at 210 min (7–8 times) and then insulin decreased back to the basal values during the rest of the post prandial period. Plasma glucose also increased rapidly after food intake (1.4–1.5 times at 210 min) and slightly decreased to the basal values along the whole remaining post prandial period. No difference between the 3 proteins sources was recorded.

We then assessed the impact of the glucocorticoids treatment on the plasma insulin and glucose levels initiated by each protein sources. After the glucocorticoid treatment and the casein meal, plasma insulin and glucose were increased during the early post prandial period when compared to the control situation. The insulin and glucose AUC were increased by 1.88 and 4.23 times, respectively **(Fig 2**). During the late post prandial period, no significant differences were observed for both plasma insulin and glucose between the control and the glucocorticoid treated mini pigs (**Fig 2**).

After the glucocorticoid treatment and the whey or the protein blend meals, a significantly higher increase of plasma glucose was also recorded during the early post prandial period **(Fig 2).** However; when compared to the casein meal, the increase in glycaemia was limited to 1.78 and 1.83 times, respectively. Contrarily to the casein meal, the glucocorticoid treatment did not generate a significantly higher increase in insulin secretion when minipigs were fed the whey or the blend protein meals (**Fig 2**).

### Plasma arterial amino acid concentrations and kinetics

#### Controls

**Fig 3:**
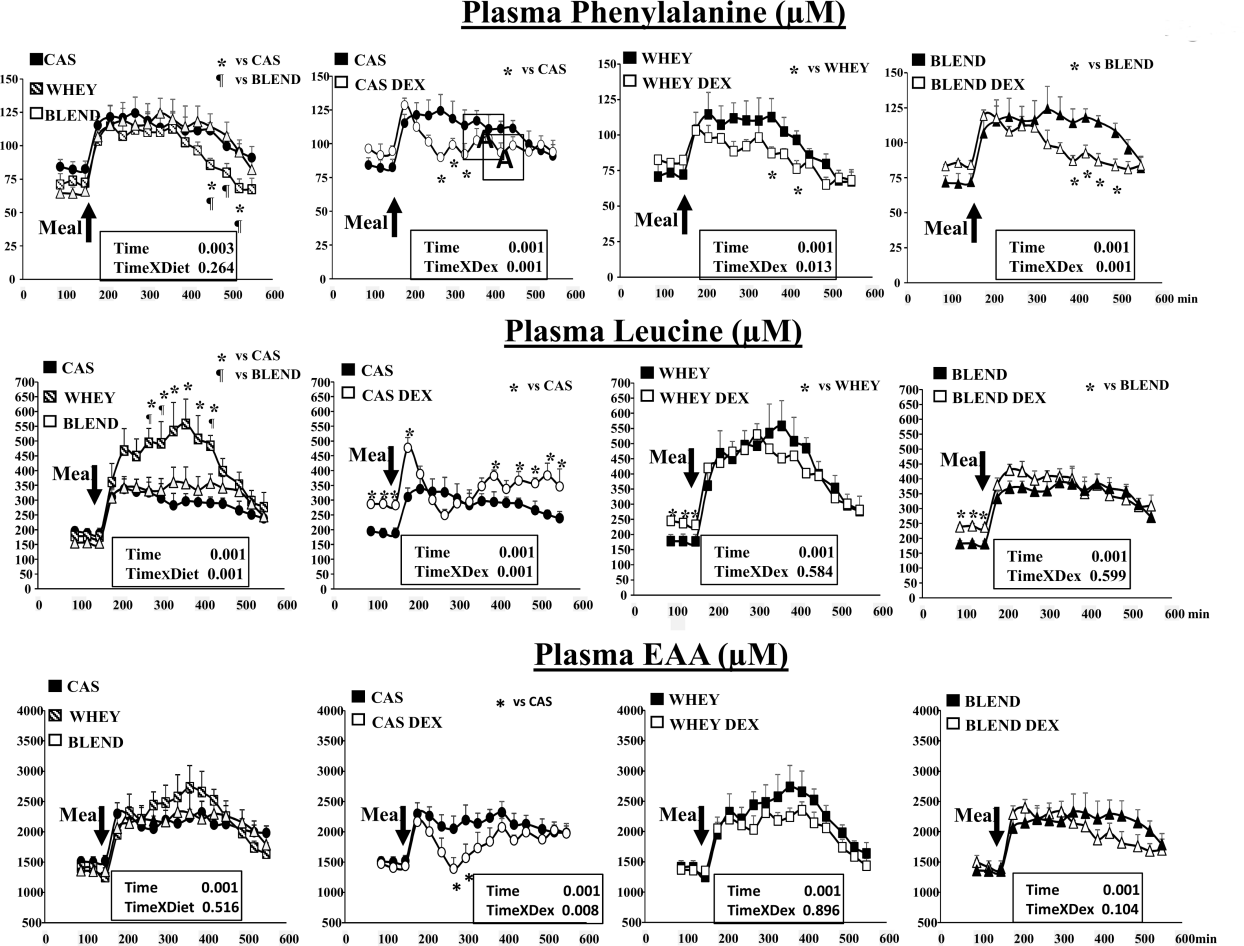
Plasma phenylalanine, leucine and total essential amino acids (EAA) in control and glucocorticoid-treated (DEXA) mini pigs receiving either a casein (CAS), whey (WHEY) or whey/plant proteins (BLEND) mixed meal. Data are presented as means ±SEM (n = 6). Repeated time variance analysis were used and at each time point, means were compared using Fischer test (for 2 means to compared). The limit for statistical difference was set at P<0.05. _*_ P<0.05 vs the control situation at the same time.

In the control groups, the effect of the ingestion of the three proteins sources on the plasma phenylalanine, leucine and EAA was described and compared (first panel of each graph line). Differences were assessed by a 2 ways ANOVA for repeated measures including time and protein sources as factors. In the control situation, ingestion of the meal was associated with a rapid and significant increase of plasma phenylalanine (**Fig 3**). Phenylalanine kinetics were very similar between the 3 protein sources in terms of maximal concentration reached after food intake but differed at the end of the post prandial period during which phenylalanine concentration decreased more rapidly when fed the whey protein meal (**Fig 3**).

Plasma leucine also increased rapidly after food intake **(Fig 3**). When fed the whey meal, plasma leucine concentrations were approximatively 2 times higher when compared to the leucine concentration obtained with the casein meal. This difference was maintained during the first two-thirds of the post prandial period to finally decrease rapidly during the late post prandial period (>400min) (**Fig 3**). When fed the plant protein meal, leucine concentrations were not significantly different from the ones observed with the casein meal during the whole post prandial period (**Fig 3**), despite the same leucine intake as the whey meal.

Regarding the total plasma essential amino acids (EAA) kinetics, no significant differences were observed after the ingestion of the 3 protein sources (**Fig 3**).

#### Glucocorticoid- treated

We then assessed the impact of the glucocorticoids treatment on the plasma phenylalanine, leucine and EAA levels initiated by each protein sources. Differences were assessed by a 2 ways ANOVA for repeated measures including time and treatment as factors. The post absorptive concentrations of phenylalanine were not modified by the glucocorticoid treatment (Fig 3). When fed the casein meal, plasma phenylalanine concentrations increased significantly in a similar way to controls during the first 30 min but then decreased rapidly to the post absorptive levels during the remaining post prandial period (**Fig 3**). With the whey and whey/plant protein meals, the phenylalanine concentrations were very similar than in controls during the first 300 min but decreased more rapidly in the late post prandial period (**Fig 3**).

Unlike the phenylalanine, post absorptive leucine concentrations were significantly increased after the glucocorticoid treatment (+35–40%) (**Fig 3**). When fed the casein meal and as observed with the phenylalanine, leucine concentrations increased during the first 30 min and then decreased rapidly back to the post absorptive levels (**Fig 3**). A slight increase of leucine concentrations could be nevertheless observed during the late post prandial period (> 330 min). In contrast, the glucocorticoid treatment did not alter significantly the plasma leucine kinetics following the whey and whey/plant protein meals (**Fig 3**).

The plasma kinetics of EAA reflected the ones observed with leucine (**Fig 3**) with a strong decrease in the early phase of the post prandial period with the casein meal and no modification with the whey and whey/plant protein meals under the glucocorticoid treatment.

### Muscle protein metabolism and balance

**Fig 4:**
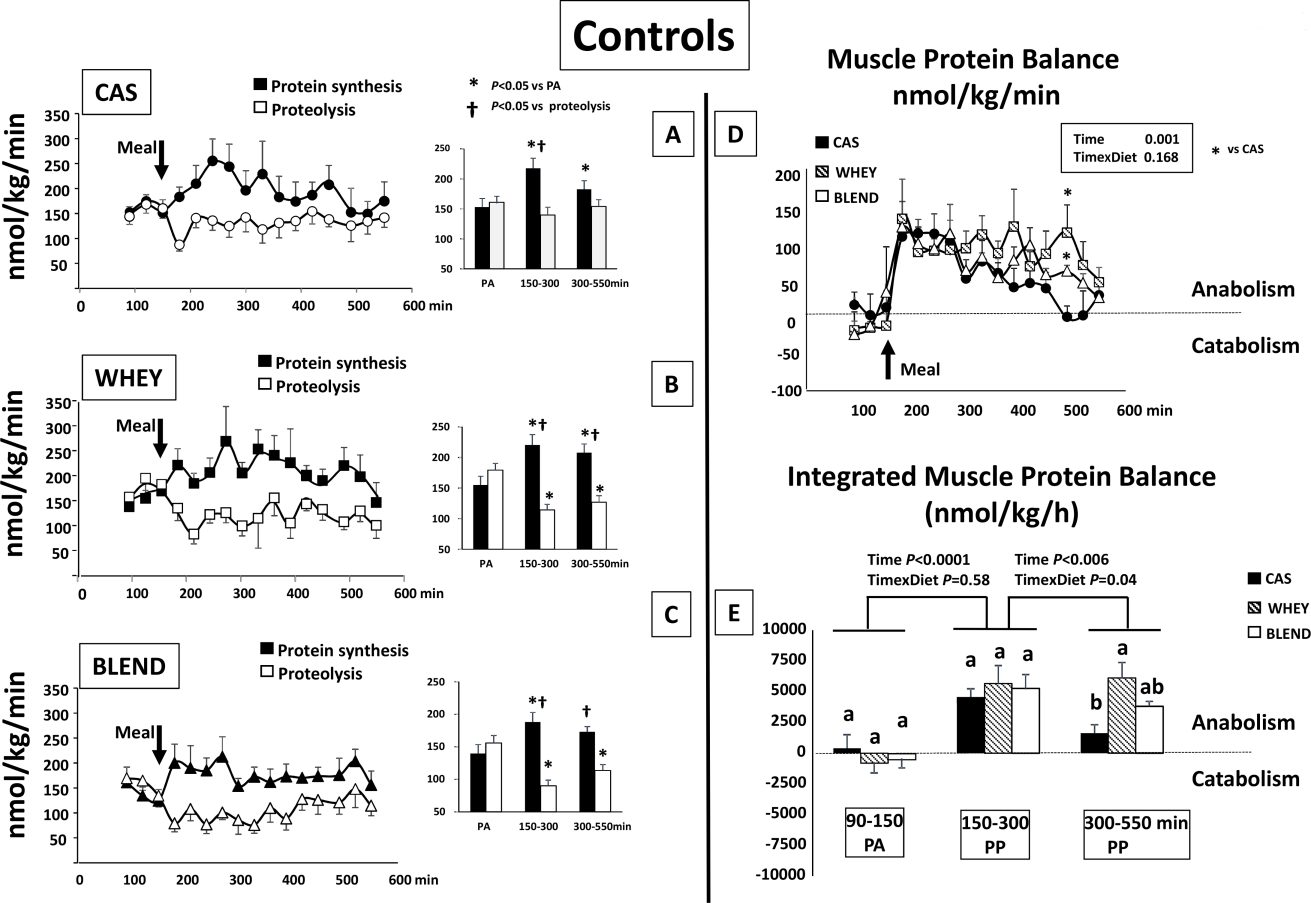
Control mini pigs: Muscle protein synthesis, proteolysis with means values for the period of time t = 90–150 (PA); t = 150–300 and t = 300–550 min (left panel) and muscle protein balance (right panel) in control mini pigs receiving either CAS,WHEY or BLEND mixed meal. Data are presented as means ±SEM. (n = 6). Repeated time variance analysis were used and at each time point, means were compared using either Tukey test (for 3 means to compare i.e integrated muscle protein balance) or Fischer test (for 2 means to compare i.e protein synthesis, proteolysis and muscle balance). The limit for statistical difference was set at P<0.05. For integrated muscle protein balance, means with different letter within each period of time are different and a global analysis between each period of time has been performed with the corresponding P value for Time and Time x Diet. PA: post absorptive; PP:post prandial.

**Fig 5:**
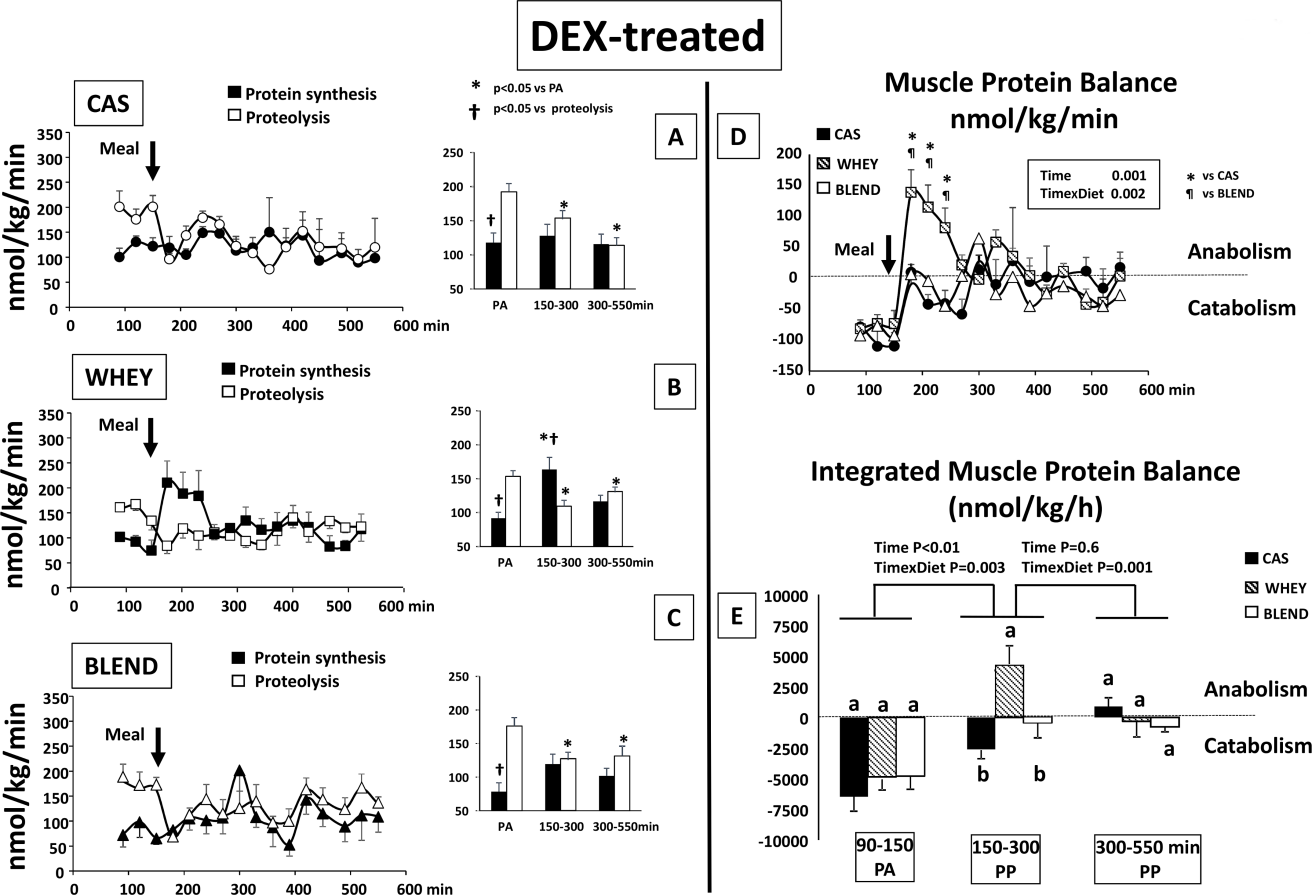
Glucocorticoid-treated mini pigs: Muscle protein synthesis, proteolysis with means values for the period of time t = 90–150 (PA); t = 150–300 and t = 300–550 min (left panel) and muscle protein balance (right panel) in glucocorticoid-treated (DEX) mini pigs receiving either CAS,WHEY or BLEND mixed meal. Data are presented as means ±SEM. (n = 6). Repeated time variance analysis were used and at each time point, means were compared using either Tukey test (for 3 means to compare i.e integrated muscle protein balance) or Fischer test (for 2 means to compare i.e protein synthesis, proteolysis and muscle balance). The limit for statistical difference was set at P<0.05. For integrated muscle protein balance, means with different letter within each period of time are different and a global analysis between each period of time has been performed with the corresponding P value for Time and Time x Diet. PA: post absorptive; PP:post prandial.

In the control (Fig 4) and the glucocorticoid-treated groups (Fig 5), the effect of each protein sources was assessed separately on muscle protein synthesis and proteolysis (panels A, B,C). Differences were assessed by a 2 ways ANOVA for repeated measures including time and metabolism (protein synthesis and proteolysis) as factors. Then we compared the protein sources effect on muscle protein balance (panels D and E). Differences were assessed by a 2 ways ANOVA for repeated measures including time and protein sources as factors.

#### Controls

In the post absorptive state, muscle protein synthesis and proteolysis were similar and muscle protein balance was balanced or slightly negative (**Fig 4**). When fed the casein meal, muscle protein synthesis increased significantly during the post prandial period with a maximal stimulation during the first 150 min whereas muscle proteolysis did not significantly decrease (**Fig 4**). As a result, muscle protein balance became positive during the early post prandial period and then decreased progressively to become equilibrated again during the late post prandial period (>300min).

When fed the whey and the blend protein meals, muscle protein synthesis also increased but by contrast to casein, a significant decrease of muscle proteolysis occurred concomitantly (**Fig 4**). Muscle protein balance became positive to a similar extend than with casein during the first 150 min. It remained significantly positive during the late post prandial period with the whey protein meal and intermediate between the whey and the casein for the whey/plant protein meal (**Fig 4**).

#### Glucocorticoid- treated

After the glucocorticoid treatment, muscle protein synthesis became lower than proteolysis during the post absorptive state and muscle protein balance was significantly negative (**Fig 5**). After food intake, a significant decrease of muscle proteolysis was observed with the 3 proteins sources during the whole post prandial period (**Fig 5**). When fed the casein and the plant protein meals, muscle protein synthesis was not stimulated and became resistant to the anabolic effect of food intake (**Fig 5**). As a result, muscle protein balance improved but remained either slightly negative or equilibrated during the whole post prandial period with the casein and plant protein meal, respectively (**Fig 5**). By contrast when fed the whey meal, muscle protein synthesis was significantly stimulated and a significant positive muscle protein balance was generated during the early post prandial period (first 150 min) **(Fig 5**). However, contrarily to the “Control” animals, this positive muscle protein balance did not last and decreased rapidly to become equilibrated during the late post prandial period (>300min).

### Muscle Akt, S6K 1 and S6 activation

**Fig 6:**
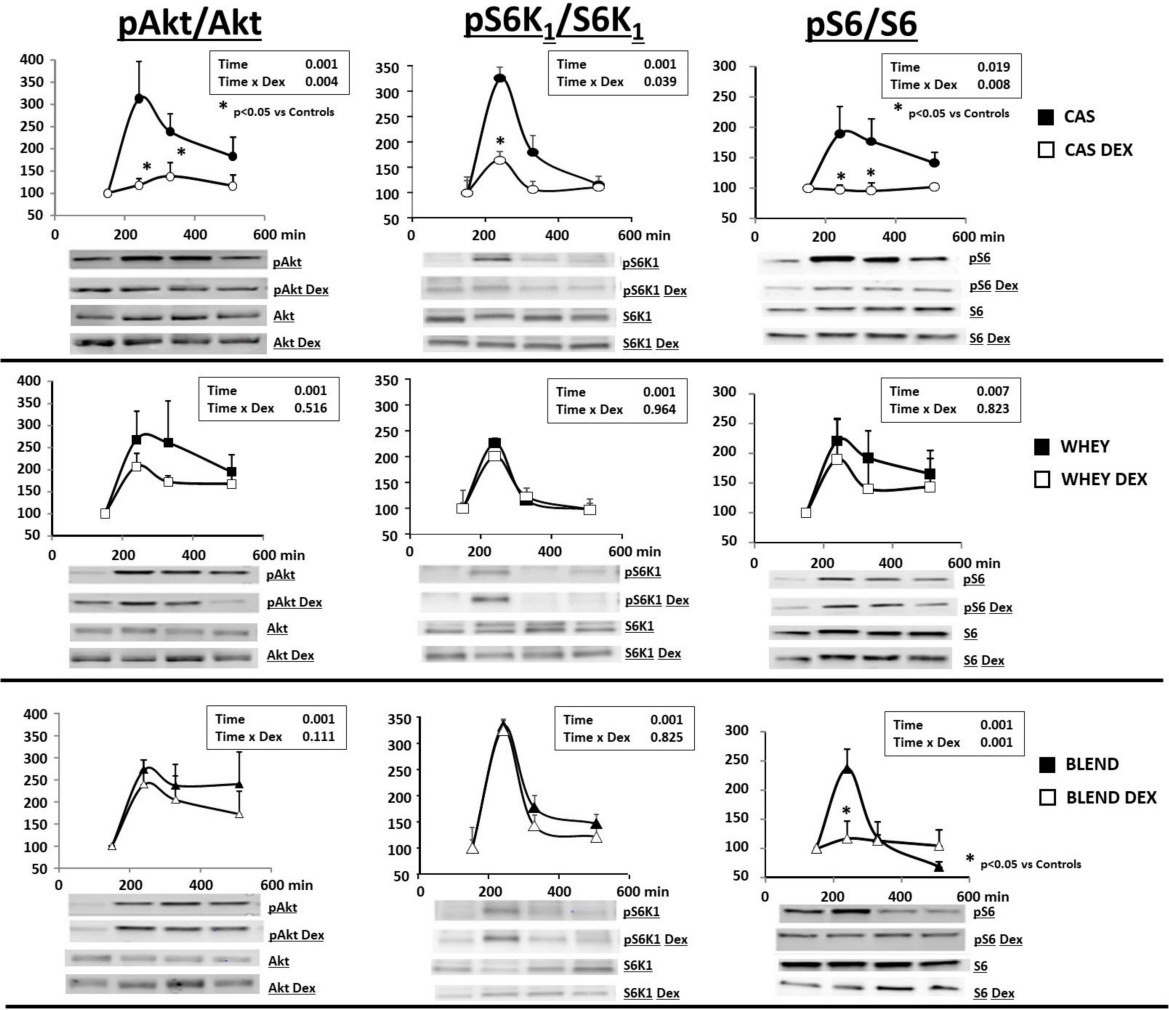
Muscle phosphorylation of Akt, S6K and S6 in control and glucocorticoid-treated (DEX) mini pigs receiving either a casein (CAS), whey (WHEY) or whey/plant proteins (BLEND) mixed meal. Repeated time variance analysis were used and at each time point, means were compared using Fischer test (for 2 means to compare). The limit for statistical difference was set at P<0.05. _*_ P<0.05 vs the control situation at the same time. Data are presented as means ±SE (n = 6) and expressed in arbitrary units (AU).

The impact of the glucocorticoids treatment on the activation of muscle Akt, 6K1 and S6 phosphorylation initiated by each protein source has been assessed and presented in Fig 6. After food intake, an increase of the phosphorylation of Akt was recorded. The maximal stimulation occurred very rapidly and reached a maximum of 3 fold for all 3 protein sources. Akt remained significantly more activated during the whole post prandial period when compared to the post absorptive state **(Fig 6)**. After the glucocorticoid treatment and when fed the casein diet, the phosphorylation of Akt was altered and was not significantly stimulated during the post prandial period (**Fig 6**). In contrast, when fed the whey or the whey/plant diets, the stimulation of Akt phosphorylation was maintained and remained not significantly different from the one recorded in control animals (**Fig 6**).

After food intake, S6K1 phosphorylation rapidly increased to be maximal at 90 min with all the proteins tested (**Fig 6**). Glucocorticoid treatment blunted the phosphorylation of S6K1 in the casein fed animals whereas it remained no altered when fed the whey or the whey/plant protein diets (Fig 6).

Regarding the phosphorylation of S6, an increase was observed after food intake (about 2–2.5 fold) within the first 90 min and was similar between all the protein sources tested (**Fig 6).** S6 remained significantly more activated during the whole post prandial period when compared to the post absorptive state except for the whey/plant protein diet for which S6 phosphorylation returned to basal values rapidly **(Fig 6**). The glucocorticoid treatment blunted the phosphorylation of S6 when animals were fed the casein or the whey/plant protein diets whereas it remained normally phosphorylated when fed the whey diet (**Fig 6**).

## Discussion

Our study clearly showed that in a catabolic situation, only whey proteins were able to initiate an anabolic response in the skeletal muscle. Despite this, the response remained transient and was only visible during the early post prandial period (i.e first 2 hours) in contrast to the 7h-stimulated muscle protein accretion obtained in healthy conditions with whey, casein or the plant-based protein blend. Despite the same leucine intake compared to the whey meal, the plant-based protein blend did not restore a positive muscle protein balance in glucocorticoid-treated mini pigs.

In healthy conditions, a significant muscle anabolic response was initiated following food intake and this response occurred whatever the protein source ingested. However, if no significant differences were recorded in the early post prandial period, a more prolonged stimulation was nevertheless observed with the whey proteins for which muscle protein anabolism between 3 to 7h post-feeding was the greatest, compared with the other tested proteins. This can be explained by the large increase of plasma leucine observed during the whole post prandial period with this dietary protein source. With whey/plant protein meal, the anabolism during this late period was intermediate between casein and whey proteins. This observation was surprising as leucine intake was identical between the whey and the plant-based protein blend and that insulin, glucose and total essential amino acids were identical between the two groups as well. However, a major difference was recorded: the leucine bioavailability remained much lower in the BLEND-fed when compared to the WHEY-fed animals despite the same leucine intake, and was not significantly different from that observed with CAS-fed animals. The reason of this discrepancy in our study is unknown, but we may hypothesize that the coagulation properties and/or the chemical behavior of the whey/plant proteins in the intestine somehow affected the digestion speed and then altered arterial leucine availability. This hypothesis is comforted by recent studies showing that the degree of gelation of the same milk protein source correlated negatively with the digestion speed and amino acid appearance in the blood stream [41, 42]. It could also result from a greater sequestration of amino acids in the splanchnic tissues [43]. Indeed, Fouillet et al. [44, 45] have shown with soy proteins a lower amino acid availability for peripheral tissues such as muscle because of their higher splanchnic extraction and conversion to urea compared to milk proteins.

The glucocorticoid treatment induced an insulin resistance as shown by the simultaneous increases in both post prandial insulin and glucose levels when fed the casein protein meal. It is not surprising as glucocorticoids are known for decades to be potent diabetogenic agents resulting from both hepatic and peripheral resistance to the action of insulin [46–48]. Interestingly, and if no peak of insulin secretion occurred in the first 30 min, an improvement of glucose tolerance was observed when fed the whey or the whey/plant proteins. This observation could be also attributed to the higher splanchnic (portal) leucine availability associated with these two diets. Indeed, leucine supplementation improved insulin-stimulated glucose transport in skeletal muscles from both adult and old rats [36, 49, 50]. Our results are in accordance with the kinetic of the intracellular signaling pathways activation involved in the stimulation of glucose transport and metabolism i.e the IRS1/PI3K/Akt pathways [51]. Indeed, muscle Akt phosphorylation occurred normally in the glucocorticoid treated animals when fed the whey or whey/plant proteins whereas it remained totally blunted when fed the casein protein meal. However, because our study was not designed to assess insulin sensitivity, these observations need to be verified with more direct measure of insulin sensitivity (e.g. using a clamp to control insulin/glucose).

In the post absorptive state, glucocorticoids treatment generated a negative muscle protein balance explained by a simultaneous decrease of protein synthesis and increase of proteolysis when compared to the control situation. Following the casein test meal, treated animals improved slightly their protein balance but they were nevertheless unable to generate a positive balance throughout the entire post prandial period. This is the typical catabolic effect of glucocorticoids already known to initiate an anabolic resistance following food intake and which could be explained by a defect in the response of protein metabolism to the dietary essential amino acids and/or the insulin resistance we observed too [15]. It is important to note that even if reduced in controls, proteolysis remained not significantly different from the post absorptive state whereas in the glucocorticoid-treated group, the difference was significantly different after the casein meal intake. The explanation could be that, in the glucocorticoid-treated group, the post absorptive values for proteolysis were increased and could be more easily detected when reduced. Furthermore, because post prandial insulin was largely increased with the casein meal in the glucocorticoid-treated group, an effect on muscle proteolysis could be nevertheless detected. Taken together, it appeared that the glucocorticoid treatment elicited an insulin resistance status on glucose metabolism, protein synthesis but not on proteolysis. More precisely, it has been shown by Rieu et al. [19] that under glucocorticoids exposure, the sensitivity of muscle protein synthesis to the anabolic effect of leucine was impaired and that the mTOR signaling pathway was altered [52, 53] and did not respond to insulin and only to very high concentration of leucine [54, 55]. In the present study, we also showed that the phosphorylation/activation of S6, a downstream effector of mTOR activation was totally blunted by the glucocorticoid treatment when fed the casein meal. More surprisingly, after a transient elevation, we also recorded with the casein meal, a very rapid decrease of the plasma essential amino acids which returned to post absorptive values to only increase again at the end of the post prandial period. Similar patterns were observed with phenylalanine and leucine. The lack of muscle anabolic response could be then explained by a simultaneous resistance of protein metabolism to leucine and a decline of peripheral leucine and amino acids availability with the glucocorticoid treatment. This decreased amino acid availability could be explained by an increase of the splanchnic extraction of amino acids. Indeed, glucocorticoids have been shown to stimulate both the synthesis of constitutive and exported hepatic proteins such as plasma proteins and albumin by 40 and 100% respectively [56]. The rapid turn-over of these proteins, associated with the low flux of dietary amino acids issued from the slow digestion of casein may explain the decrease of peripheral amino acids we observed in the present study.

Interestingly, with the whey and plant protein blend, this decreased amino acids availability with the glucocorticoid treatment was only slightly visible at the end of the post prandial period for phenylalanine and not present at all with total essential amino acids and leucine. Despite this, only whey proteins were able to generate a positive muscle protein balance whereas the whey/plant proteins remained, like casein, unable to overcome the anabolic resistance initiated by the glucocorticoid treatment. This difference was in accordance with the activation status of the muscle mTOR signalling pathway which was able to be activated by whey but remained ineffective in stimulating the downstream S6 phosphorylation after the whey/plant proteins test meal. The reason of this discrepancy could be explained by the difference in leucine concentrations reached with the two dietary proteins (Fig 3). Indeed, it has been postulated that the stimulation of muscle protein synthesis could significantly occur if leucine concentrations were sufficient to reach a definite anabolic threshold [1]. With glucocorticoid treatment, this anabolic threshold was increased (anabolic resistance) and only the higher levels of bioavailable leucine generated by the whey proteins were able to exceed this threshold and then elicited an anabolic response. Nevertheless, it remained transient and only occurred in the early post prandial period (i.e. first 2 hours) whereas whey proteins stimulated muscle protein accretion for almost 7h in healthy conditions. Recently, it has been shown that muscle protein synthesis stimulation after feeding is of finite duration even if amino acids availability is still increased and mTOR signalling pathways still activated [57, 58]. It has been postulated that muscles can sense they are “full” and this phenomenon has been named the “muscle full” effect [59, 60]. In our study, despite a similar amino acids/leucine availability and a similar activation of the mTOR signalling pathway after whey protein ingestion, the duration of the anabolic response was shortened by glucocorticoids evidencing their role in a premature 'muscle-full' response. Such phenomenon has been previously hypothesized to occur in muscle wasting conditions [59]. However, in milder anabolic resistance states like in old adult subjects, it has been shown that plant proteins when ingested at same leucine content that whey, may be as efficient as animal proteins to overcome an anabolic resistance [61]. Depending of the plant protein source, it remained to be studied if this would not require a too much increase of dietary protein intake if not mixed with animal proteins.

In conclusion, our study showed that in healthy conditions, a muscle anabolic response was initiated whatever the protein source ingested. However, even if a plant/dairy protein blend was adjusted to the same leucine intake than a whey protein source, the plasma leucine availability was lower probably due to food matrix effect and/or increased splanchnic extraction of amino acids. These hypotheses merit to be explored in future studies, as in the context of sustainable protein production systems for human nutrition in the 2050 horizon [62], an increased proportion of plant protein-based ingredient in the human diet is recommended. In catabolic situations associated with an anabolic resistance after food intake, only the whey protein diet was effective to initiate an anabolic response. However, it remained transient and not effective enough to prevent muscle wasting unless the duration of this anabolic response could be optimized by other means.

## Abbreviations

PA: post absorptive
Akt: protein kinase B
AUC: area under the curve
CAS: casein
CHO: carbohydrates
DEXA: dexamethasone
GC-MS: gas chromatography–mass spectrometry
p-Akt: phosphorylated protein kinase B
PP: post prandial
p-S6: phosphorylated ribosomal protein 6
RDA: recommended dietary allowances
S6: ribosomal protein 6
BLEND: whey/plant proteins blend
